# How to make the rhetoric of joined-up government really work

**DOI:** 10.1186/1743-8462-5-22

**Published:** 2008-11-04

**Authors:** Jim Hyde

**Affiliations:** 1 Department of Human Services, GPO Box 4057, Melbourne 3001, Victoria, Australia; 2 Centre for the Study of Ethics and Medicine in Society, Monash University, Melbourne, Victoria, Australia

## Abstract

"Joined-up' government and 'whole-of-government' approaches have evolved over the past two decades from the simple 'one-stop-shop' concept to much more formal organisational structures mandated at the highest levels. In many cases, the participants in these developments were learning on the job, as they responded to community and political demands for better service delivery and more accountability. This paper looks back at some of those developments and proposes a schema to assess and place policies, strategies and programs.

## Introduction

Over the past fifteen years governments in a number of modern democracies have moved toward an integrated approach to policy development and service delivery. The United Kingdom [1] experience most fully illustrates the concept but it has also been taken up in other European countries, Canada and New Zealand as well as Australia. These approaches have many factors in common across disparate systems and sectors. Central to them all are concepts of government as facilitator, collaboration between sectors, and partnerships with civil society and communities. The outcomes depend on values and underlying political ideologies, on social conditions and cultural appropriateness or acceptability, and on the willingness of leaders and partners to engage with ideas. The language of policy has evolved differently depending on the research or theories on which it is based. The context of policy making for 'joined-up' government is a complex system layered with multiple sectors and levels of decision-making. This leads to a significant problem with confusion across sectors about what the rhetoric really means at a practical level. The author's involvement at a number of levels on some of these developments has led to the development of a schema to assess and place policies, strategies and programs. Its aim is for use by those working with governments, program developers and service providers in improving policy and program development, and service outcomes. The schema is built on the emergence of theories for 'joined-up' government or 'whole-of-government' approaches, as well as developments in leadership and organisational theory and recognises the hierarchical structures of government within sectors (vertical), but also the need for collaboration across sectors (horizontal). The key players at each level may be formally within government, or influencers on government.

This paper aims to outline the key factors within the schema, and aims to assess its role in the success of collaboration and partnership. In doing so it will consider the conditions and factors for, and the barriers to, successful implementation. The schema developed has four levels of decision-making and action: Government (Strategy); Bureaucracy (Policy and Program Development); Organisational (Program Management); and Local (Service Delivery). At each level, strategic themes for engagement have been identified along with implementation issues and processes. These point to the factors and conditions for success as well as the barriers that can be expected to the implementation of collaboration and partnership. The development of such a schema is built on the emergence and theories of 'joined-up' government or "whole-of-government" approaches, as well as developments in organisational theory and leadership. It is also informed by debate on the relation between evidence and policy – can policy be evidence based or evidence informed? The paper concludes by showing how the schema can assist both the debate and the practical implementation of policy by assisting policy makers and service providers in making more informed judgements with appropriate authority.

## Background

A brief review illustrates the breadth of the theories that have informed 'joined-up' government. The key issues raised are a) the strategic themes for engagement, b) the implementation processes that have been adopted and c) using evidence to inform policy.

### Strategic themes for engagement

The theory of organisational development in both the public and private sectors has converged over the past fifteen years. Morgan [2], Kotter [3] and Wheatley [4] built on the earlier management theory developments and applied them in a way that was relevant for both the public and private sectors. In public sector theory Hyde [5] and Pollitt and Bouckaert [6], for example, extended and redefined theories for the public sector. The Australian Commonwealth, States and Territories governments (in common with many governments in similar countries) have various programs and projects that have tested models of 'joined-up' government. An evaluation of some of these was undertaken by Success Works for the Institute of Public Administration Australia (IPAA) [7] and informed the schema development along with the emergence of work on community capacity building and resilience. Other work undertaken by Szirom and Hyde [8] also informed its development. In the US, DeSeve and Lucyshyn [9] identify public value networks that are similar to whole-of-government networks in Australia. Theories of community capacity have also emerged [10-12]. For communities, community capacity building focuses largely on sustainability of capacity, programs that promote it and quality service delivery and on the development of resilience among individuals, families and communities.

### Implementation processes

As whole-of-government or 'joined-up' government processes have developed, a number of matters have been central to the connections that are seen as necessary. These relate not only to capacity building, both at a community and organisation level, but to community and individual resilience, and social connectedness. Taking these concepts from theory through policy to implementation is a key challenge. Capacity building is now seen as a key to successful 'joined-up' government initiatives that lead to sustainable action [11,13]. There are five main domains in which community agencies and organizations must have capacity if they are to engage in successful partnership: Organisational Development; Workforce Development; Resource Allocation; Leadership; and Partnership.

#### Social Capital

Putnam [14] attributes to social capital "features of social organization, such as networks, norms, and trust, that facilitate coordination and cooperation for mutual benefit". He points out that social capital is an essential ingredient in both economic development and effective government through networks of civic engagement that promote accepted norms of community and individual reciprocity, promote coordination, cooperation and information about trustworthiness in others; and provide examples of collaboration that become cultural norms of behaviour. Szreter and Woolcock [15] provide a comprehensive analysis of social capital development that synthesises bonding, bridging and linking social capital, arguing that explicit recognition of them is required in successful Public Health interventions especially where they are based in partnerships.

*Resilience *(which is central to many theories of community capacity as well as social capital) is generally regarded as the capacity to recover from adversity and difficult or harmful events. There is also an inherent capacity to defend or protect oneself from such events. Resilience encompasses individual through to community capacities. It is an important concept in the schema because of the assumption in much of the policy and program development of 'joined-up' government that individuals and communities can take levels of control that will lead them to rebound from deprivation, previous withdrawal of services and alienation. This is especially the case in indigenous policy and programs, but is also present in engaging communities with long-term unemployment, and rural deprivation. Benard [16] describes four key domains of the personal skills or resources required for resilience: social competence, problem solving skills, autonomy and a sense of purpose and future. Social competence can be broadly characterised by qualities including responsiveness, flexibility, empathy and caring, communication skills, a sense of humour, and any other prosocial behaviour. Problem solving skills include the ability to think critically, abstractly and reflectively and to develop alternative approaches to cognitive and social problems. Autonomy is derived from the capacities of self-esteem, self-efficacy, internal locus of control and adaptive distancing. Sense of purpose and future includes attributes of health expectancies, goal directedness, success orientation, achievement motivation, educational aspirations, persistence, hopefulness, hardiness, belief in a bright future, a sense of anticipation, a sense of a compelling future and a sense of coherence.

#### Connectedness

Underlying the concept of resilience is the need for 'connectedness', meaning that individuals have a sense of belonging with their family, their peers, their school and the wider community. This is achievable through socialising processes that include perceived opportunities to participate in activities within the social unit; the amount of interaction and involvement with others; the extent of positive reinforcement of involvement and interaction; and emotional, cognitive and behavioural skills for involvement and interaction that enhance reinforcements and perceptions of reinforcement. Socialising processes (interactions, involvement, reinforcements and skills) help the individual to form bonds or attachment to others in the family, peer or community unit, based on the values, norms and behaviours of other people in those environments. It is noted that *the establishment of prosocial bonds to family and school are seen to be predictive of prosocial behaviour *[17]. Prosocial bonds are viewed as protective factors inhibiting development of antisocial or 'at risk' behaviours. These factors are also central to theories of social capital, and in health are also central to responses to health inequalities and strategies for equity in health outcomes.

### Using evidence to inform policy

Finally, the use of 'evidence' in the development of policy – its adoption by policy makers and decision makers – is a key issue. There has been a growing interest in concepts of evidence based on evidence informed policy over the past few years. Bowen and Zwi [18] have reviewed this literature and described how evidence is transformed in an "adopt, adapt, act" context. This involves a) sourcing evidence, b) using that evidence in policy making, and c) recognising the capacity in the system for implementing policies informed by the evidence. They have developed a framework for action for 'evidence-informed' policy that includes such factors as the context in which policy is being made (e.g. cultural, economic, resources, system development); the factors that influence decision-making (e.g. usefulness, level of influence of individual or organisation); the different models of policy-making (e.g. political, knowledge-driven, problem-solving); and the factors that determine capacity to implement (e.g. stated objectives, expertise, resources at individual, organisational and system levels).

It is important that a schema for strategic action built from these developments reinforces the knowledge that has been generated in practice over the past two decades. The schema draws from the literature the major identified conditions, factors and barriers for successful action so that in planning intersectoral, collaborative partnerships strategies can be developed to enhance prospects for success and sustainability.

### The Schema

There are four levels at which solutions that lead to action must be developed:

**1. Government **(Strategy)

**2. Bureaucracy **(Policy and Program Development)

**3. Organisational **(Program Management)

**4. Local **(Service Delivery)

**Figure 1:**
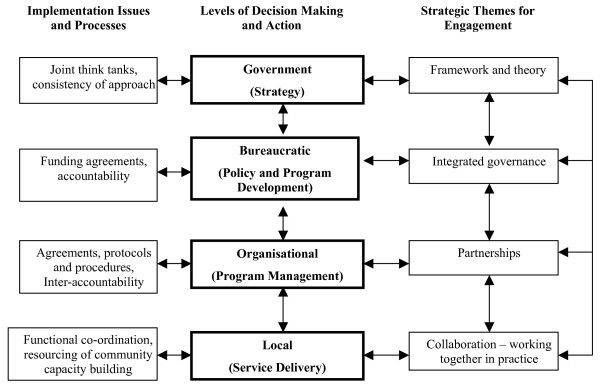
Conceptual schema for strategic action.

While there are differences between the Australian Federal, State and Territory governments in the detail of specific aspects of their public administration, the similarities are significant and the framework reflects how they tend to organise their administrative activities. Figure 1 summarises the various levels for strategic action and sets against these the major themes for strategic development [on right] and some possible issues for practical action [on left].

#### 1. The Strategy Level (Government)

The *strategy level *is government and concerns high level policy formation and the conceptualisation of policy in terms of theoretical frameworks and strategic directions. This includes Ministers and their advisors, the political parties that support them, consultants/think tanks that they engage, and senior Departmental advisors. In 'joined-up' government the levels of influence between these players and the context in which they exercise that influence will be important considerations. This is the political level of Government where strategic directions are decided with a broad political policy. Political leadership at this level can be a crucial factor in success.

#### 2. The Policy & Program Development Level (Bureaucracy)

At the *bureaucratic level *are the forms of departmental organization and processes through which policy is implemented. These include policy development and organisational structures. A whole of government approach must co-ordinate the contribution of various departments into a coherent and integrated response. This usually requires inter-departmental committees and inter-departmental processes. One of the key issues that will emerge here is that of accountability and how, in a vertical, hierarchical set of structures, individual Ministerial and Departmental accountability can be mediated. Specific policy development at this level will be informed by and based in strategic political policy from the Government level as well as evidence from the program and local levels, and from research.

#### 3. The Program Management Level (Organisational)

At the *program management level*, that is, the level of organization that develops programs from policy and directs service delivery programs, the issue is how to manage contributions across sectors. The capacity of each of the sectoral players must be identified as well as their level of influence especially when at this level there will be a range of formal government organisations and non-government stakeholders.

#### 4. The Service Delivery Level (Local)

At the *local level*, the service delivery level, the key question is how collaboration between community services and agencies can be effected on the ground. This is especially important where the services in question will range across a number of formal government agencies, different levels of government and non-government agencies. At each level, a series of conditions, factors and barriers have been identified as the schema has developed. These reflect the input of a number of workshop participants from a range of settings and are illustrative not exhaustive.

##### Using the Schema

Within this context networks for 'joined-up' government may cross the levels or be focussed in them. They may also range across different agencies including non-government partners. This reflects the complexity of the issues for joined up responses. However, the importance of the schema is for locating the authority for decision-making so that the outcomes of networks for 'joined-up' government are sustainable. It is important to note that while the levels of the schema equate to levels at which decisions will be made, in practice many decisions will be made across levels and indeed across sectors. The qualifier is that the authority for decisions will rest in a particular level so that while accountability may suggest a hierarchy, practice allows for devolution of decision making through providing a mandate for action or delegation of authority. The schema should not be seen to be hierarchical other than in an accountability sense. It will in practice allow for collaborations across sectors and across levels so that, for example, a program could be the product of a number of stakeholders from the local level in government and non-government services providing service and outcome data to planners at the program level who are working with program developers at the bureaucratic level. In this sense the flow of information especially about implementation including the development of evidence, and evaluation will be two-way.

### The Government (Strategy) level

#### The mandate for policy direction, program development and action resides at this level

The conditions that underpin the schema at the Government level may be the most difficult to develop and maintain as they rely on the political will of governments and the vagaries of public opinion. Nevertheless, as the volatility of the electorate has become more pronounced governments have reacted in two ways – first, by pursuing openly populist policies, and second, by providing the conditions for 'joined-up' government. This provides the mandate for action. The factors that facilitate the mandate flow from these conditions.

There are a number of *conditions *promoting success at this level. First, leadership capacity, the ability of government to make decisions that have long-term effects, is important if a mandate to breach organisational silos is to be gained. (Lead Ministers must have leadership capacity to persuade colleagues if barriers at other levels are not to emerge.) Second, an explicit policy commitment is necessary because the success of program development at other levels will depend on maintaining policy support. Third, resource commitment is essential because long-term resources have been identified as a key condition for successful 'joined-up' programs along with the ability to share and/or pool funds. Fourth, an understanding of evidence and knowledge is an important condition at this level especially the interaction between evidence and decision-making, and of the way in which different actors will understand and accept evidence. Finally, a commitment to stakeholder consultation is needed with stakeholders and communities involved in different ways that inform decision-making. This will have important flow-on effects at other levels.

The *factors *that are needed at this level are negotiation skills and capacity at political level, preparedness to collaborate across sectors and boundaries, recognition of opportunities and barriers, and Preparedness to adopt, adapt and act on evidence in policy context. These factors appear to be critical to success. They rely on network support, access to senior decision makers and the capacity to sustain resources. There are also significant *barriers *that must be recognised including the political cycle and public attitudes. Recognising political cycles and the capacity of government to act decisively depending on community attitudes are important if these barriers are to be overcome.

### The Bureaucratic (Policy and Program Development) level

#### The authority for prioritisation of policy and resources resides at this level

Capacity in the public sector provides the conditions for successful collaboration. While the mandate for action is based at the Government level, the authority to act on that mandate lies at the senior levels of the bureaucracy. 'joined-up' governance requires a capacity in the public sector beyond public policy and program development, including also flexibility and accountability capacity. The factors that flow from these conditions are directly supported by the factors and conditions at the Government level.

The *conditions *include a public policy capacity. There is variable capacity in policy making in many government agencies. Formal training in public policy and frameworks within which decision-makers can develop public policy and programs from political policy are essential elements of success. Second, program development capacity, the ability to translate policy to achievable programs, requires a capability and knowledge in issue content, leadership and management, and in community awareness. Third, flexibility in policy development and understanding the variety of information that will inform policy development, interpreting that information and assessing achievability are key issues for this condition. Fourth, evidence translation capacity such as the "adopt, adapt, act" model is a useful tool to translate various forms of evidence and to interpret information that might not reach the gold standards of scientific or clinical evidence. Fifth, accountability of decision-making, especially transparency in decision-making, will ensure that contestable outcomes are more likely to be acceptable, and will improve the capacity for a policy or program to achieve its goals.

*Factors *identified at this level are knowledge and commitment of public policy development, ability to recognise and translate evidence, commitment to collaboration and partnership, and high level funding agreements together with infrastructure support and commitment. Having the technical and political skills to translate government policy to public policy and broad programs are important factors that must be maintained and improved within organisations. The *barriers *at this level are departmental silos, a lack of relevant data, lack of flexibility in funding arrangements, and poor understanding of performance indicators. Organisational capacity is often undermined by inflexibility within departments, by conflicting accountabilities and data requirements, and different understandings about evaluation and indicators. These issues should be tackled at the beginning of 'joined-up' work if they are not to undermine successful outcomes.

### The Organisational (Program Management) level

#### The authority for implementation of policy and expenditure of resources resides at this level

The Organisational level is the operational domain of the public sector. It has the authority to implement policy and programs mandated by government and authorised by policy made in the bureaucracy. It is the level to which funding is devolved and from which operational funds are expended. The conditions for success at the Operational level depend on capacities of management and administration. The factors that apply are also operational.

The *conditions *at this level are first, program management capacity, which is essential at this level if successful implementation is to occur. Second, flexibility in policy development. Making broad government programs fit local initiatives and conditions requires an understanding of the "adopt, adapt, act" model and an ability to recognise how local conditions will affect outcomes. Third, flexibility in service development, because while existing service development may already fit proposed policy and program directions, it may also be able to be adapted to achieve and promote flexible outcomes. Fourth, capacity to implement evidence-informed policy decisions, where the "adopt, adapt, act" model provides a tool that will enable the underlying evidence on which policies and programs are built to inform developments at this level. Finally, accountability of decision-making through transparency. This should encourage the return to decision-makers of local evidence and information that will complete a virtuous circle to improve chances for successful outcomes and sustainability.

The *factors *include a commitment to collaboration and partnership, an ability to adapt evidence and knowledge to practical application, a capacity to link funding agreements and performance agreements, an ability to develop agreements, procedures and protocols, and an understanding of relevant performance indicators. Excellent administrative capacity combined with local knowledge and leadership improves the chances of successful achievement. Good technical skills should be combined with an understanding of broader conditions and factors. The *barriers *are lack of relevant data, lack of flexibility in funding arrangements, poor understanding of performance indicators, and a mismatch between policy objectives and program outcomes. Lack of technical skills and assistance, inadequate data and information, and poor capacity to translate policy objectives into program development require attention as work proceeds at this level.

### The Local (Service Delivery) level

#### The authority for flexible service delivery and expenditure of resources resides at this level

Programs, the delivery of services and operational collaboration occur at the Local level. The conditions that underpin success rely on the authority devolved and the flexibility with which local services can be package and delivered. They also rely heavily on appropriate and adequate funding. The factors that attach at the Local level include good understanding of the local environment and a commitment to collaboration.

At this level the *conditions *are first, and importantly, the authority to act. The local managers must have a clear authority to act or to be aware of the levels of their authority is local implementation is to be successful. Second, the capacity to implement policy and program objectives because local level understanding of policy development and program development is necessary if translation to specific implementation is to be achieved. Third, adequate resources for processes are a key condition for success. Local implementation will require adequate resources for management and evaluation, and for data/information collection, management and reporting. Fourth, adequate resources for implementation of services with an important understanding at this level that local implementation should be made to fit the level of resources available, which could mean that staged or partial implementation is undertaken. Finally, a capacity for evaluation and development of evidence. In a successful local implementation a virtuous circle of data collection will provide evaluation and the building of evidence for further development.

The f*actors *at the local level include commitment to functional collaboration and coordination, an understanding of the local environment, a capacity to recognise local knowledge and transform it through evaluation, and a capacity to manage local resources. In particular, local workforce skills and capacity are essential and investments may need to be made to compensate or build capacity in for example, partnership development, and planning. At the local level there must be a clear understanding of the boundaries in the commitment to a policy and program if it is to be implemented successfully. This will be balanced by a capacity to assess appropriate evidence to complete a virtuous circle of implementation, evaluation and further development. The accompanying *barriers *include inadequate management processes, inadequate, inconsistent and inflexible resources, and importantly (and commonly) a mismatch between local demand and policy objectives. Lack of capacity to manage, inflexible or inadequate resources and an inability to convince local communities of the importance of proposed outcomes will present barriers that require concerted effort between the local and organisational levels.

**Figure 2:**
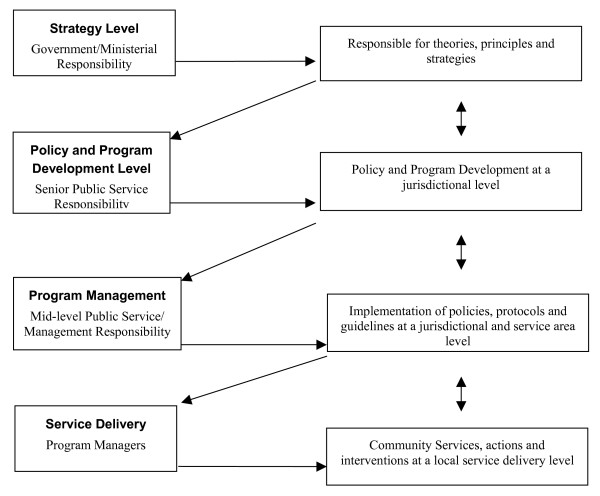
Levels of responsibility for strategic action.

Figure 2 shows the levels of responsibility for action that flows from the schema. The mandate for each level derives from its accountability upwards.

### Developing and Testing the Schema

The schema has not yet been rigorously tested although it has been tested in the field by the author. It has evolved during completion of a range of work where it was employed at different stages to develop the conditions, factors and barriers, and to test proposals under development. The importance of linking the conditions for success between the levels is central to the understanding of participants at all levels. Even though those using the schema may be concentrating on only one or two aspects, their understanding of where a mandate is provided and at which point the authority for action is placed is important for robust and sustainable decision making. This should lead to better program development based on informed policies, and more effective service delivery. As noted above the conditions, factors and barriers identified at each level of the schema reflect the input of a number of workshop participants from a range of settings and are illustrative not exhaustive. When using the schema to guide workshop discussion, participants should be encouraged to identify those that are relevant to their own situations and build strategies based on them. The schema can be used prior to planning and implementation to test that roles and responsibilities at each stage are clear, or during implementation and in evaluation to solve emerging problems or test outcomes.

The schema has been presented in workshop at an international conference where a number of suggestions were made for refinement. Participants from a number of national perspectives were supportive of the framework the schema provides. It has also been used in workshop situations a number of times during its development. The workshops were at various levels including an international study tour group of senior health officials, an Australian workshop group of senior human services officials and staff of non-government organisations. In each case workshop participants have used the schema in conjunction with capacity building exercises or strategic planning exercises. The outcomes from these presentations also suggest that the schema is useful in a number of settings and is not limited only to central government policy and program development, or to the Australian situation. Health officials from a developing nation found it useful in reviewing a change from a centralised system to decentralisation, and it could also be used within an organisation to identify levels of authority and capabilities to develop and implement organisation policies in specific programs.

## Conclusion

The conceptual schema was developed through a number of projects undertaken by Dr Tricia Szirom and the author over a number of years from work that began with policy development in NSW Health in the late 1990s and for the Australian Government in 2000–01. The author refined it over the period from 2001 through policy development in a major learned medical college and in workshops. This paper aimed to outline the schema and to assess it role in building successful collaborations and partnerships. It has identified key factors and conditions at each level as well as barriers to success. It has discussed the use of the schema in some settings where it contributed to discussion of the practical implementation of policy and program development. The author is grateful for comments made at various stages of development by workshop participants, and later by senior policy makers and other colleagues. It is offered as a schema for locating decision-making in for example, planning, resource allocation or program implementation, especially where whole-of-government or 'joined-up' interventions and activities are being planned.

### Competing interests

The author declares he has no competing interests
